# Qualification of the Vitrimeric Matrices in Industrial-Scale Wet Filament Winding Processes for Type-4 Pressure Vessels

**DOI:** 10.3390/polym17091146

**Published:** 2025-04-23

**Authors:** Jonathan Alms, Anna Katharina Sambale, Jannick Fuchs, Niklas Lorenz, Nina von den Berg, Tobias Conen, Hakan Çelik, Rainer Dahlmann, Christian Hopmann, Markus Stommel

**Affiliations:** 1 Institute for Plastics Processing in Industry and Craft, RWTH Aachen University, 52074 Aachen, Germany; 2 Institute of Polymer Materials, Leibniz-Institute for Polymer Research Dresden e.V., 01069 Dresden, Germany; sambale@ipfdd.de (A.K.S.);; 3 Institute for Materials Science, TUD Dresden University of Technology, 01062 Dresden, Germany

**Keywords:** vitrimers, vitrimeric resin, 4-aminophenyl disulfide, carbon fibre reinforced polymers, carbon fibre composites, thermo-mechanical properties

## Abstract

The production of fibre-reinforced composites for use in applications such as type-4 pressure vessels for hydrogen storage is achieved through the use of a thermoset matrix. However, the recycling of thermosets presents a significant challenge due to the lack of established recycling methods. Epoxy-based vitrimers show thermoset characteristics during the manufacturing and utilisation phases but exhibit thermoplastic behaviour at elevated temperatures of 190 °C. This study investigates the industrial-scale production of carbon fibre reinforced vitrimers via a wet filament winding, as exemplified by a type-4 pressure vessel demonstrator. Processing conditions of industrial processes have yet to be applied to vitrimers; therefore, two vitrimer formulations are compared to a conventional epoxy thermoset. The processability and resulting composite quality of wound composites using these materials as matrices are compared. The mechanical properties of the composites are compared using an interlaminar shear strength test, demonstrating that the vitrimeric matrices exhibit 19.8% (23 °C) and 49.2% (140 °C) improved interlaminar strength. Consequently, the epoxy-based vitrimers investigated in this study can be employed as a direct replacement for the thermoset matrix in industrial-scale applications, with the potential for recycling the composite. To increase composite qualities, the winding process must be adapted for vitrimers, since a pore free composite could not be achieved.

## 1. Introduction

Wet filament winding is a well-established industrial process commonly used to manufacture composite-wrapped pressure vessels, such as those for hydrogen storage, pipes and drive shafts [1,2,3,4,5]. This process is valued for its high material throughput, the ability to achieve a high fibre volume fraction, and its flexibility in realizing different fibre orientations [2,6]. Pressure vessels for hydrogen storage are typically reinforced with continuous carbon fibres embedded in epoxy-based thermoset matrices due to their long-time stability and resistance against thermal loads during cyclic hydrogen loading [4,5,7]. However, a significant challenge arises at the end of the vessels’ lifecycle due to the permanent molecular bonds within the thermoset matrix, which hinder recyclability [8]. Vitrimers have recently gained attention as a promising alternative to traditional thermosets, offering the potential for repair, reuse and recycling (3R) [9,10,11,12,13]. Vitrimers possess dynamic molecular bonds within their network, allowing for reconfiguration after curing. This unique property enables mechanical recyclability and fibre recovery at the end of the product’s life [14,15,16]. However, the dynamic reversible bonds which are needed to allow recyclability also limit the thermal stability of the material, which could lead to premature failure especially during the loading of the pressure vessel [17,18]. Due to their recent introduction to the polymer processing field, components made of vitrimers have so far been limited to specimen-sized parts, with or without a reinforcement phase [19,20,21,22,23]. These parts are typically manufactured through the hot pressing of imine- and ester-based vitrimers, as well as through the initial scalable production of cured vitrimer prepregs using disulfide-linked epoxy vitrimers [20,24,25,26,27]. Bio-based vitrimers derived from starch, cellulose or sugars, as well as saturated cardanol retrieved from cashew nut shell oil, are discussed to reduce the environmental impact of vitrimeric materials even further while offering good mechanical properties [28,29,30,31]. Curing agents, such as aromatic 4-aminophenyl-disulfide (4-AFD) bears a disulfide bond, which can undergo associative dynamic bond exchange at elevated temperatures. Its functional amino end groups enable curing with epoxy groups identical to established curing mechanisms [32,33,34,35,36]. This compatibility with standard epoxy resins allows for the use of well-established production processes, as demonstrated in a previous study [32]. However, industrial-scale production of fibre-reinforced vitrimers remains underexplored, primarily due to the currently limited availability and high cost of vitrimeric curing agents.

This paper investigates a wet filament winding process using epoxy-based vitrimers utilizing a 4-AFD curing agent in an industrially scalable process in the production of type-4 pressure vessels, since the application of vitrimers in industrial processing remains scarce [37,38,39]. Therefore, we demonstrate the commercial applicability of epoxy-based vitrimers in current products and manufacturing techniques. In order to qualify the resin for service temperatures for applications in type-4 pressure vessels (up to 80 °C), the thermal properties of the neat epoxy vitrimer are compared to a reference thermoset to analyse the matrix behaviour at common temperatures in the application (Section 3). Additionally, the thermo-mechanical properties are compared via a tensile test to analyse the material behaviour of the unreinforced matrix at different temperatures (Section 4). Two vitrimer matrices and one reference thermosetting epoxy resin are processed in a wet filament winding process, and the resulting composite quality is determined using microscopy images of the cross-section (Section 5). Finally, the composites are compared using tensile tests and interlaminar shear strength tests, showing a higher strength of the composite with a vitrimeric matrix in comparison to the reference due to more ductile behaviour and higher stiffness retention at high temperatures (Section 6). This qualifies the epoxy-based vitrimer as a possible replacement for conventional thermosetting epoxy resins in industrially scalable processes such as the production of type-4 pressure vessels with the benefit of 3R properties.

## 2. Determination of Thermal Behaviour

### 2.1. Material Formulation and Curing Process

Within a previous study, the epoxy resin EPIKOTE Resin 04976 provided by Westlake Epoxy GmbH (Duisburg, Germany) was qualified for a filament winding process with two different types of curing agents [32]. To qualify the resin curing with a vitrimer curing agent, the 4-AFD, Molekula Limited (Darlington, UK) for the filament winding process, the rheologic and kinetic material properties are compared to the material characteristics of a resin with a conventional anhydride curing agent (EPIKURE Curing Agent 04976) and a catalyst (EPIKURE Catalyst 04976). Three material combinations are mixed according to the producers’ recommendations or preliminary works (Table 1) [32,40].

The viscosity of the mixed vitrimeric curing agent and resin (V04976) is much higher (~100 mPas at 80 °C) at identical temperatures compared to the RE (~40 mPas at 80 °C) since the 4-AFD is crystalline at room temperature and has a melting temperature of 84 °C, thus, being highly viscose at 80 °C [32]. Since the viscosity of the material in the resin bath during the filament winding process is one of the most important processing parameters to ensure proper fibre impregnation, the temperature is adjusted by a 20 °C increase for the vitrimeric mixture during processing compared to RE [3,41]. In addition, a highly refined version (a small molecular weight distribution) of the EPIKOTE Resin 04976 and the EPIKOTE Resin 0162 is also tested. In conventional winding applications, the EPIKOTE Resin 0162 is used to adjust the viscosity of the 04976 resin to lower values, while maintaining the epoxy equivalent and identical reaction mechanisms according to the suppliers suggestion [40]. Here, the EPIKOTE Resin 0162 + 4-AFD is used as is, not in combination with EPIKOTE Resin 04976, as it would usually be for industrial purposes, to reduce the processing viscosity as much as possible. Since the curing processes and the reaction kinetics are identical to the EPIKOTE Resin 04976, the same weight ratio of resin and curing agent are applied for both (Table 1). The curing reaction is performed in an oven at 90 °C for 1 h followed by 140 °C for 2 h, according to preliminary works, reaching almost 99% degree of cure [32].

To manufacture non-reinforced, resin-only specimens by curing, the resin is preheated to 80 °C for 60 min before mixing with the curing agent (and catalyst for RE). For the RE material, the resin, curing agent and catalyst are mixed in a Speedmixer (DAC 400 FV, Hauschild SpeedMixer Inc. (Hamm, Germany) for 1 min at a weight ratio of 100:80:1. V04976 and V0162 are processed with the same parameters but at a weight ratio of 100:43, respectively, and without using any catalyst. The mixed resins are then transferred into moulds with dimensions of 130 × 110 × 1 mm^3^ and cured at 90 °C for 1 h, followed by post-curing at 140 °C for 2 h in a temperature-controlled oven (UF450 plus, Memmert GmbH + Co.KG (Schwabach, Germany) equipped with Type-K thermocouples fixed on the mould to monitor the temperature during curing and post-curing [32]. The procedure reaches a fully cured state of all materials, which is ensured by the absence of additional reaction heat during the heating in thermal tests. Therefore, this curing procedure reaches a fully cured state of all the material combinations. After curing and post-curing, the moulds are cooled to room temperature, and the produced plates are demoulded. Specimens required for mechanical testing are subsequently milled from the plates.

### 2.2. Determination of Topological Freezing, Glass Transition and Decomposition Temperatures

The loading and unloading of pressure vessels for hydrogen storage lead to inhomogeneous thermal buildups resulting in the need for temperature stability of the matrix material [5]. The glass transition temperature (T_g_) of the cured epoxy resin is typically used to describe a product’s application range in terms of temperature stability [42]. Vitrimeric materials show a second transition temperature, the topological freezing temperature (T_v_) [43,44,45]. T_v_ determines the temperature above which rapid molecular bond exchange can occur, resulting in polymer flow and a phase change from a viscoelastic solid to a viscoelastic fluid [46]. It has been demonstrated that the majority of vitrimers relying on aromatic disulfides exhibit a T_v_ located well below T_g_ [47,48]. Therefore, the glass transition represents the decisive criterion for designating a service temperature range for projected applications. Below T_g_, the polymer structure is essentially frozen, restricting any bond exchange [49], and only the canonical glass transition can stop the vitrimer from undergoing irreversible shape changes [50]. First, thermogravimetric analysis (TGA) is used to determine the decomposition temperatures T_d_, establishing an upper limit for the thermal loads applied during subsequent thermal and mechanical testing.

#### 2.2.1. Determination of Decomposition Temperatures

**Figure 1:**
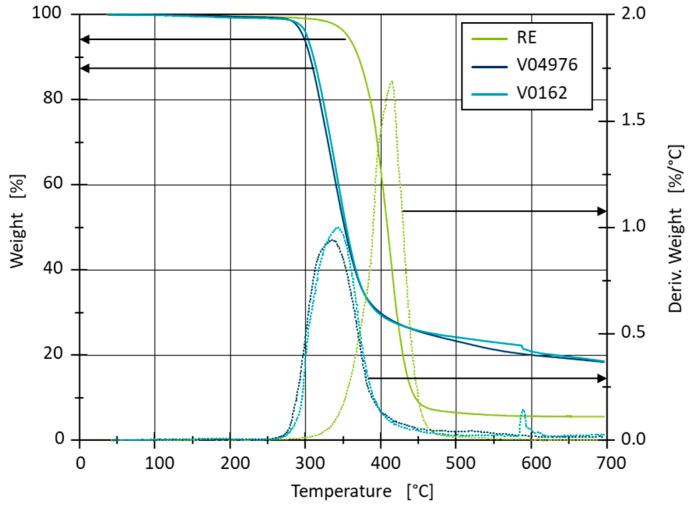
Thermal stability of RE, V04976 and V0162 from TGA measurements with the first derivative of the weight.

A Q500 system from TA Instruments Inc. (New Castle, DE, USA) is used for the thermogravimetric analysis. The non-reinforced resin samples are produced and fully cured, as stated in Section 2.1. The samples are heated from room temperature to 700 °C at 10 °C/min in an inert N_2_-atmosphere. The weight loss and the rate of weight loss of the three materials with temperature are used to determine the precise start of the decomposition as well as a possible multi-stage decomposition (Figure 1).

The decomposition curves of both vitrimers are similar, with the material V0162 losing mass slightly later. Due to the wider molecular weight distribution of the 04976 resin in comparison to the 0162 resin, the degradation of the share of smaller molecular weight could lead to the earlier degradation onset in the V04976. The decomposition of the vitrimers begins at 250 °C. At approx. 590 °C, a local maximum occurs with V0162, which is presumably an artefact of the measurement. The RE decomposition begins at 300 °C and shows a higher rate of decomposition in comparison to the vitrimers. The weight reduction of the vitrimers slows down from 400 °C, leaving 18% ash residue at 700 °C, where the RE decomposes almost completely. The high residue indicates a large number of inorganic components in the vitrimer 4-AFD curing agent. In order to prevent the decomposition of the materials during further testing, a maximum temperature of 230 °C cannot be surpassed without considering decomposition.

#### 2.2.2. Estimation of Transition Temperatures

**Figure 2:**
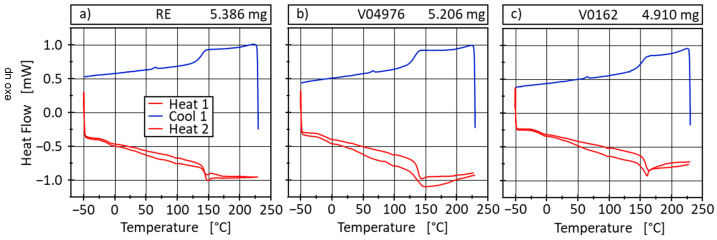
DSC measurements of RE (**a**), V04976 (**b**) and V0162 (**c**) containing two heating (10 °C/min) and one cooling (10 °C/min) cycle in-between and isothermal holding phases (2 min) at T_min_/T_max_.

Differential scanning calorimetry (DSC) measurements are carried out using a Q2000 DSC, Waters/TA Instruments Inc., Austin, USA, to calorically determine the amount of heat required for physical transformations of the materials and to determine the transition temperatures T_g_ and T_v_ precisely. Both the heat flow and temperature sensors of the DSC Q2000 were calibrated with sapphire and indium standards, and a baseline correction was also performed beforehand. The three fully cured materials are measured according to the TGA-findings in the temperature range of −50 to 230 °C in order to prevent decomposition. A heating rate of 10 °C/min is applied and the measurements are performed in an inert N_2_-atmosphere (Figure 2). At −50 °C and 230 °C, an isothermal holding phase of 2 min is added to the measurement method in order to equilibrate the samples at the beginning and end of the cooling and heating phases. At approximately 100 °C in the heating curves and at approximately 70 °C in the cooling curve, artefacts can be detected reproducibly in all samples, which presumably originate from the appliance and are neglected in the analysis.

The glass transition temperature areas of the materials are determined using the half-height method according to DIN EN ISO 11357-1 [51] from the second heating cycle (Table 2).

For all materials, a β-transition is observed in the two heating curves in the range of 0 °C, which describes the start of the side group mobility in the material. The V04976 shows a slightly lower T_g_ in comparison to RE. However, the V0162 shows a significantly higher T_g_ of 158 °C than the other two materials. The V0162 should, therefore, show the least reduction in mechanical properties if heated to T_g_ of the conventional material formulation (RE), which is in accordance with findings in the following interlaminar shear strength test performed on composite samples in this study (see Section 5.2). This should allow all materials to be used up to 100 °C in application before a significant reduction in mechanical properties must be considered. All materials are, therefore, usable to withstand temperatures found at loading hydrogen in type-4 pressure vessels [5].

At temperatures at around 200 °C, an exothermic effect occurs in all cooling curves which cannot yet be clearly explained since all samples are fully cured prior to the measurement. It is possible that this effect nevertheless indicates post-curing or incipient decomposition, or, with a minor probability, an artefact from the transient settling of the DSC. This effect is more pronounced with the two vitrimer types than with RE, which can be attributed to the slower amin-reaction between the epoxy and the 4-AFD in comparison to the anhydride reaction occurring with the conventional curing agent EPIKURE curing agent 04976. Supporting post-curing is the reduction in the exothermic effect in the second heating cycle. However, no significant curing peak is visible, indicating a high degree of curing prior to the measurement.

The thermal analysis using DSC does not show any transition determining T_v_ at the tested temperature range. Therefore, no thermally induced phase transition into the viscoelastic fluid phase could be determined and a dissociation of the disulfide bonds cannot be expected at temperatures below 230 °C. We assume a net-zero heat flow of the dynamic exchange reactions occurring in that temperature interval. However, the disulfide bond also allows for associative exchanges, which can occur at lower temperatures and long-time scales, allowing for a gradual change in network morphology [43,44,52].

#### 2.2.3. Demonstration of the Vitrimeric Properties via Compression Moulding

Since the thermal investigations do not indicate the existence of T_v_, only T_g_ is observed in the thermal analysis, and the vitrimeric properties of the resins with the vitrimer curing agent 4-AFD will be demonstrated by reversible forming at temperatures beyond T_g_ using the associative disulfide exchange. Since the state of a vitrimeric melt comparable to thermoplastic melts is not reached, the disulfide exchanges require an additional mechanical force applied in a compression moulding process, invoking a network morphology change and permanent shape alteration.

For this purpose, the materials V04976 and V0162 are moulded into a rectangular shape and cured, as mentioned in Section 2.1. After curing and cooling, the specimens are heated in a convection oven and shaped using a wave-shaped tool. Due to the difference in T_g_, both vitrimers are shaped at 80 K above T_g_, resulting in different temperatures and additional masses to simulate compression moulding in a controlled environment at very low pressures. The samples have a rectangular cross-section and a size of 40 × 4 × 2 mm^3^ (L × W × H). The test procedure can be found in Table 3. The temperatures and masses were determined empirically in preliminary tests.

**Figure 3:**
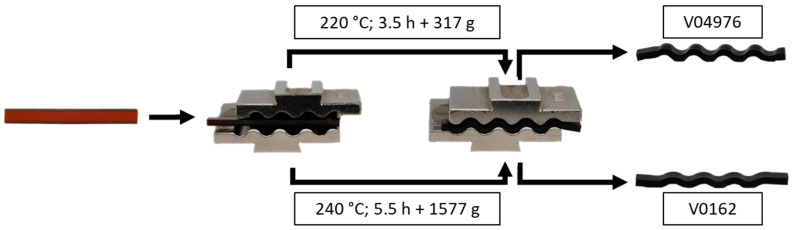
Demonstration of the vitrimeric properties of V04976 (**top**) and V0162 (**bottom**) using reshaping tests under temperature and pressure.

Figure 3 shows that the vitrimers took on the waveform after the compression moulding tests, so the vitrimeric behaviour of the materials is confirmed and a permanent reshaping is achieved without crack formation, which would be expected with conventional thermosetting material. After reshaping, both materials showed a black, non-transparent coloration (right), compared to the brownish, clear initial state (left). This can indicate thermally induced oxidation processes, which is in accordance with the TGA measurements, since the temperatures are held for >0.5 h to homogenise the temperature in the tool and the specimen.

The V04976 specimens are shaped with a low mass and at 220 °C, showing a permanent deformation after cooling (Figure 3). The V0162 specimens show, with higher pressure (weight) and higher temperatures, a less pronounced degree of deformation, although both temperatures are chosen at ~80 °C above the glass transition temperature.

The results clearly show potential for subsequent reforming and proves the vitrimeric properties through the presence of associative disulfide exchanges. However, the material response at high temperatures and under loads show differences, which are characterised further by thermo-mechanical analysis.

## 3. Comparison of Thermo-Mechanical Properties of Vitrimers and Conventional Thermosetting Epoxy Resin

The thermo-mechanical properties of the non-reinforced resin systems are determined in uniaxial tensile tests to assess the mechanical performance of each material at room temperature and 80 °C. For this purpose, plates of all three materials are cast in a mould and cured, as described in the materials section (see Section 2.1). The plates are then milled into tensile bars with geometry 1BA, following DIN EN ISO 527-2 [53], and subsequently tested using a Universal Tester 1456, Zwick/Roell (Ulm, Germany).

**Figure 4:**
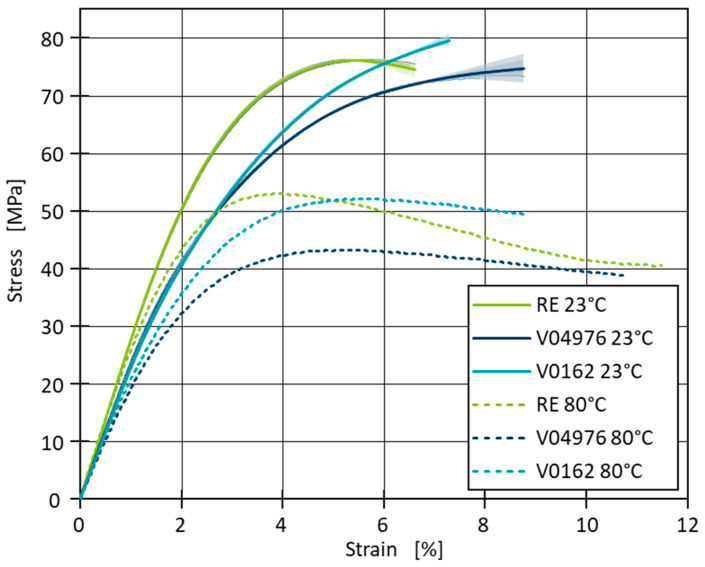
Averaged results of the stress–strain curves for 23 °C (averaged results) and 80 °C testing temperature for the uniaxial tensile tests.

The specimen elongation is measured with a tactile extensometer, MultiXtense, Zwick/Roell (Ulm, Germany) and the force is determined using a 1 kN load cell. The specimens are clamped using a pneumatic clamping system at 8 bar, with a gauge length of 55 mm, a parallel length of 45 mm (for the 1BA specimen geometry), and an extensometer gauge length of 25 mm. The tests are conducted at a speed of 1 mm/min, corresponding to a strain rate of 4%/min relative to the extensometer gauge length. A climate chamber from Zwick/Roell (Ulm, Germany) is employed to control the ambient testing temperature. At 23 °C, three samples are measured, and the results are averaged (Figure 4). When the temperature increases to 80 °C, only one sample is measured for each resin system. The tests are all analysed in accordance with DIN EN ISO 527-2. All materials show a typical elastoplastic behaviour, with RE exhibiting the highest initial stiffness and the highest tensile stress at break, as shown by the steep slope in the elastic region followed by a maximum stress of ~76 ± 0.2 MPa. Materials V04976 and V0162 follow a similar trend but reach slightly lower maximum stress values of ~73 ± 1.3 MPa and ~77 ± 2.2 MPa, respectively. The strain at break varies, with RE showing the lowest and V04976 and V0162 showing higher elongations at break.

**Figure 5:**
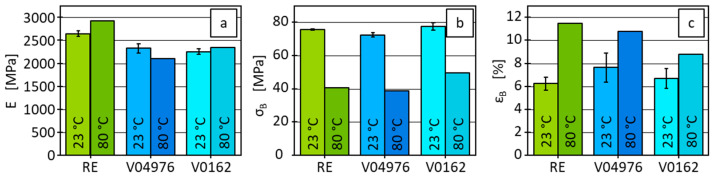
Results of Young’s modulus (**a**), tensile stress at break (**b**) and tensile strain at break (**c**) of RE and both vitrimers for 23 °C (averaged results with standard deviation) and 80 °C testing temperature.

At 80 °C, the materials exhibit a reduction in strength, as indicated by the lower maximum stress and stress at break values. Regarding the stress at break, RE fails at ~41 MPa, while V04976 and V0162 also experience reductions in stress at break to ~39 MPa and ~49 MPa, respectively (Figure 4). Furthermore, the materials show a more pronounced plastic deformation, with increased strain at break, particularly for materials RE and V04976, where the stress–strain curves deform further before breakage. The slope of the initial linear-elastic region, which corresponds to Young’s modulus (E), is also noticeably less steep at 80 °C, reflecting the reduction in stiffness (Figure 5).

Young’s modulus shows a minor increase for material RE when the temperature is elevated from 23 °C to 80 °C, with values of 2640 MPa and 2920 MPa, respectively. In contrast, the other two materials, V04976 and V0162, show only slight differences in stiffness at 80 °C compared to 23 °C. This indicates that for RE, the material becomes slightly stiffer at higher temperatures, while this trend is differing for V04976, where the stiffness slightly decreases and for V0162, where it marginally increases. Nevertheless, these results must be viewed with caution, as only one sample was tested at 80 °C and, therefore, only hints at these trends can be regarded. The tensile stress at break $\sigma_{B}$ reveals a significant decrease at 80 °C for all three materials (Figure 5). RE shows a reduction from 76 MPa at 23 °C to 41 MPa at 80 °C, whereas material V0162 drops from 77 MPa to 49 MPa. Material V04976 is characterised by a similar trend, reducing from 73 MPa to 39 MPa. This behaviour indicates a pronounced thermal softening effect, where higher temperatures lead to a considerable reduction of strength across all materials. The tensile strain at break $\varepsilon_{B}$ demonstrates an increase in ductility for all materials when tested at 80 °C. RE shows a notable rise from 6% to 11%, while V04976 increases from 8% to 11%. V0162 also exhibits increased elongation, rising from 7% to 9% (Figure 5). These results suggest that while the materials lose strength at higher temperatures, they become more ductile, allowing for higher deformation before failure.

The observed effects can be explained by the distinct network morphologies and thermal properties of the materials, particularly considering that all tests were conducted below T_g_ and T_v_. The RE material, a permanently crosslinked thermoset, exhibits high stiffness and strength at both 23 °C and 80 °C due to its rigid network, with a high decrease in strength at the elevated temperature. The vitrimers V0162 and V04976, which rely on dynamic covalent disulfide bonds, show a significant increase in elongation at break at 80 °C. However, since the tests are performed below the temperature, where permanent deformation is demonstrated, the dynamic bond exchange mechanisms are not in the dissociative regime, and the increased ductility is likely due to the thermal softening of the polymer chains rather than associative exchange driven network rearrangement due to the small time scale of less than 3 min in comparison to the compression moulding test where time scales are in the magnitude of hours and at much higher temperatures. The reduction in tensile stress at break in the vitrimeric samples at 80 °C is attributed to increased chain mobility, which weakens the material’s resistance to mechanical stress without causing significant bond reconfiguration.

With the conducted measurements, vitrimeric properties of the material combinations V04976 and V0162 are demonstrated. The thermo-mechanical properties of the vitrimers show a comparable behaviour to RE below T_g_, a temperature at which the dynamic nature of the covalent vitrimeric bonds should have no effect on mechanical properties. Considering applicability of the investigated vitrimeric resin in context of type-4 pressure vessels, a less stiff matrix of the fibre-reinforced wrapping should facilitate better load distribution and, therefore, to higher interlaminar strengths, which are validated on winded composites.

## 4. Comparison of Wet Filament Winding Process Using Vitrimer and Thermoset Matrices

To demonstrate the potential of industrial-scale production of parts with a vitrimer matrix, a wet filament winding process of a type-4 pressure vessel is demonstrated. Due to the current market price of the 4-AFD, we limit our investigations to the straight main body. In this study, the straight main body without the vessel caps is produced, resulting in a carbon fibre reinforced plastic (CFRP) pipe with three of the previously studied matrix materials RE, V04976 and V0162. As a result, only one fibre orientation in the circumferential direction was achieved. Pipe segments with different matrix materials are compared via tensile tests and interlaminar shear strength to evaluate the performance and the possibility of using vitrimeric matrices as a substitution for the thermosetting reference.

### 4.1. Production of CFRP Pipes

In the production of the CFRP pipes, a robotic filament winding setup is used, with the relevant process parameters resulting in a stable process shown and described in previous works [54,55]. Figure 5 shows the machine setup producing a CRFP pipe with a vitrimer matrix system. To ensure sufficiently low viscosity of the material, the resin components, as well as the vitrimer curing agent, are preheated to 90 °C in a vacuum oven UT 62000 of Hereaus (Hanau, Germany). The material is mixed evenly by stirring and dosed directly into the impregnation bath of the winding system.

**Figure 6:**
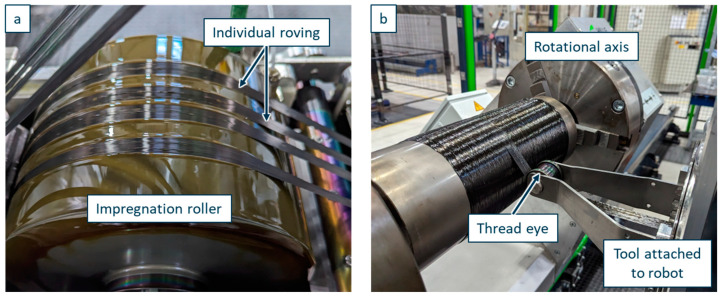
Industrial-scale production of CFRP with vitrimeric matrix material. (**a**) Roller for filament impregnation with V04976. (**b**) Deposition of hoop layers to produce a pipe shaped sample.

To compare the results of the wet filament winding process using a vitrimeric matrix to the wet filament winding using a conventional thermoset matrix (RE-material combination), all three material formulations are processed. The RE matrix is processed at 60 °C, resulting in a viscosity of ~80 mPas [32], with a doctor blade gap of 300 µm and a fibre band force of 20 N. As previous investigations show, successful processing occurs with a designated impregnation bath temperature between 60 and 80 °C, which is in accordance with the manufacturer [32,40,54,55]. These process parameters lead to a uniform resin coating of the impregnating roller and, thus, an average resin impregnation of the roving. The carbon fibre used is an ITS 50 F23 24K with 1600 tex purchased from Teijin Carbon Europe (Heinsberg, Germany). The winding speed is 0.2 m/s, winding six consecutive hoop layers into a composite pipe with an inner diameter of 146 mm (Figure 6). Both resin systems (V04976 and V0162) with vitrimeric curing agents were processed with the same machine parameters, with the only adjustments being the resin bath temperature according to previous research to 80 °C, resulting in a viscosity of ~100 mPas (V04976) [32] in order to compensate for the difference in resin viscosity. The resin (EPIKOTE 0162) of the V0162 is used as a dilutant for the EPIKOTE 04976, therefore, resulting in a lower viscosity at the same processing temperature.

Since the viscosity during production is visibly higher with both vitrimeric matrices due to cooling until deposition, an additional pipe with fewer laminate layers and the V0162 matrix is manufactured with an elevated impregnation bath temperature of 90 °C in order to compare production quality at elevated temperatures and even lower viscosity. This additional CFRP pipe is only tested regarding its impregnation quality using microscopic analysis.

### 4.2. Visual Comparison of Fibre Impregnation Quality

**Figure 7:**
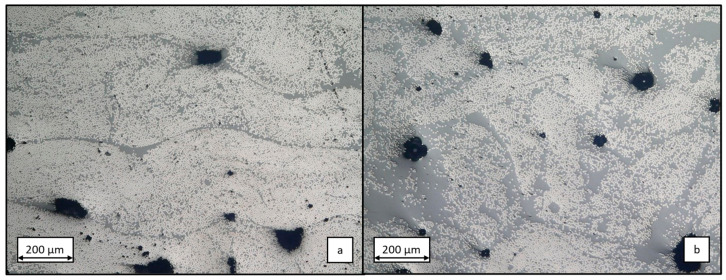
Representative microsection of the wound test specimens made with RE matrix (**a**) and V04976 matrix (**b**) at 200× magnification.

The impregnation quality is defined by the fibre volume content (fvc) and the pore content (pc). To quantify these values, randomly selected cross-sections measuring 10 × 2 mm^2^ are examined at 200× magnification using the reflected light microscope VHX-600, Keyence (Osaka, Japan). Figure 7 presents a comparative analysis of exemplary micrographs of samples produced with the RE matrix (Figure 7a) and V04976 matrix (Figure 7b). Both images show a similar pore content. However, less densely packed fibres in the V04976 specimen indicate a lower fvc, which is supported by slightly thicker cross-sections. The quantitative comparison reveals that the laminate manufactured with a RE matrix exhibits a more compact structure, despite the same fibre band tension during winding, but the higher viscosity led to more matrix pickup and less matrix squeeze out. The roving configuration from the winding process can also be observed in the RE sample, whereas the additional matrix content in the V04976 sample does not allow visual separation of each roving. The porosity of both samples is similarly high in comparison but varies considerably over the entire circumference of the samples.

**Figure 8:**
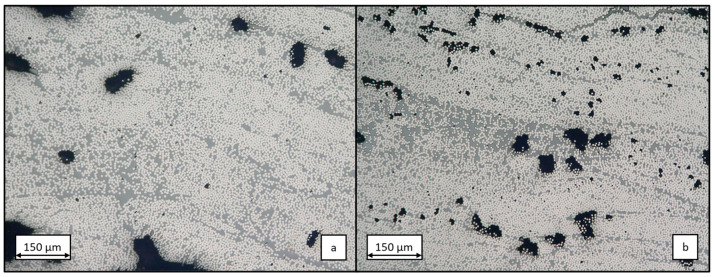
Microsection of wound test specimen made from vitrimer resin 0162 at processing temperature of 80 °C (**a**) and of 90 °C (**b**) at 300× magnification.

The influence of viscosity reduction during the winding process is evident in the comparative microscopy images presented in Figure 8. Figure 8a depicts a microsection of the test specimen manufactured with V0162 at an impregnation bath temperature of 80 °C. The comparatively high proportion of pores with large pores and a low proportion of resin between the individual filaments is attributed to the suboptimal impregnation quality of the roving in the winding process. A direct comparison with the additional produced CFRP pipe with V0162 at a higher processing temperature of 90 °C and, thus, lower viscosity, reveals a reduction in the size of the individual pores present and the formation of more resin-rich regions in the laminate (Figure 8b). It can be concluded that higher processing temperatures result in an improvement in impregnation quality, although the winding process used in the study does not achieve pore-free production.

**Figure 9:**
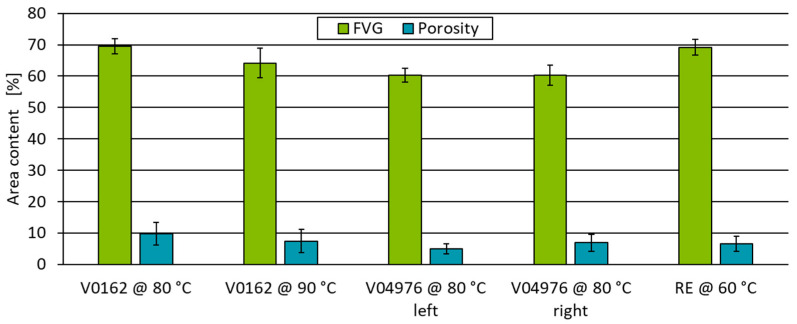
Fibre volume content and porosity of specimen analysed using WekaSegmentation plugin. Area contents are calculated on a sample size of 30 per test point.

A quantification of fvc and pc is conducted in six respective cross-sections using the open-source image analysis software ImageJ (Fiji V2.9.0) with the WekaSegmentation (V4.0.0) plugin [56]. Based on a classification of a training dataset, the images are divided into fibres, matrices and pores according to their grey scale values within a set threshold and analysed according to the respective area fractions (Figure 9). The reference process using the RE matrix, a fvc of 69.2 ± 2.6% and a pc of 6.5 ± 2.4% is evaluated. During the curing of the matrix in an oven at 140 °C, the vitrimeric pipe sample V04976 was slightly tilted, resulting in an uneven matrix material distribution, which is shown in the reduced porosity on the left-hand side of the pipe. Therefore, a sample at the end (V04976 (left)) and the start of the pipe (V04976 (right)) are evaluated, showing the same fvc, but a reduced porosity at the end of the pipe (Figure 9). Additional testing using higher processing temperatures during the filament winding process will be addressed in future research, this study focusses on the properties of the specimens with V04976 matrix processed at 80 °C and V0162 matrix at 80 °C.

The results of the impregnation quality suggest that the tensile strength and flexural strength of the RE are increased compared to those of the vitrimer matrix specimens due to the comparably high fvc combined with lower porosity for V0162 (Figure 9 V0162 @ 80 °C) and the higher fvc compared to V04976. In addition, the higher Young’s modulus and higher strain at break measured with the non-reinforced RE-system double up for a superior composite behaviour in comparison to the samples with a vitrimeric matrix.

## 5. Mechanical Comparison of the CRFP Pipe Segments

Since the resulting composite morphology, characterised by fibre volume content and porosity through distribution and size, also determines the resulting mechanical properties due to fibre/matrix bonding—which cannot be investigated by imaging techniques—mechanical testing is conducted at this point to determine the macroscopic strength properties. To determine the strength properties, pipe ring segments with a nominal width of 10 mm are prepared and tested using a split-ring test (ASTM D2290a [57]). This test qualifies a good fibre/matrix adhesion if a high percentage of the nominal fibre strength is reached. The higher pore content in the vitrimeric specimens resulting from not impregnated fibres or voids cannot distribute loads and, therefore, result in reduced strength. The composite is further evaluated by quantifying the matrix/fibre interaction using an interlaminate shear strength test performed in accordance with DIN EN ISO 14130 [58].

### 5.1. Analysis of Mechanical Strength of the CRFP Pipes

**Figure 10:**
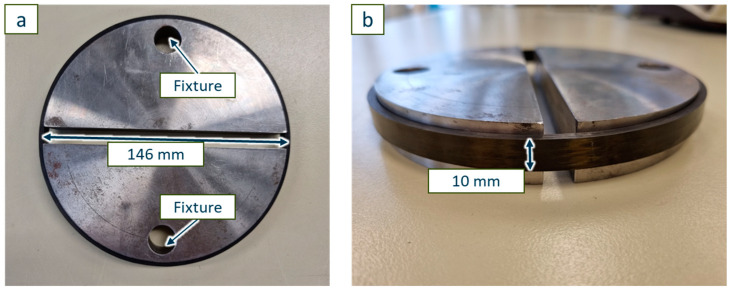
Split-ring test setup with two half discs ((**a**) top view) and the mounted 10 mm wide carbon fibre reinforced vitrimer ring ((**b**) side view).

In the split-ring test, two halved disks of the same diameter as the inner diameter of the pipe segment (146 mm) are used to support the sample while the tensile test is performed (Figure 10) [57]. This allows a force application in the fibre direction without bending the ring at the points of the fixture. The comparison of the resulting tensile strength is used here to initially qualify the winding process with successful fibre impregnation and good interlaminar adhesion. During the winding process, fibres were deposited only in the radial direction with an angle of 88.7°. The tensile strength of a pipe segment can be compared to the theoretical tensile strength of the fibres by applying a small angle simplification in order to identify the fibre/matrix bonding. A high percentage of fibre strength, therefore, indicates a good fibre/matrix bonding and a low impact of not impregnated fibres.

The split-ring test is performed on six ring segments per material distance controlled with a speed of 1 mm/min on a universal testing machine Z150 from ZwickRoell GmbH & Co. KG, (Ulm, Germany). Every sample of each material shows the same fracturing behaviour at breaking point. This allows for a direct comparison of the sample strength of the material combinations. From the fibre content used during production and the force applied just before failure, the mean strength of each material combination can be determined (Table 4). The measured strength is compared to the theoretical fibre strength stated in the data sheet of 5100 MPa to determine a good fibre/matrix bonding. Approximately 90% of the fibre strength is reached, showing that all material combinations are able to adhere to the fibres, no unhindered unwinding is observed and the effect of the difference in measured porosity seems to be negligible.

The comparison of the reached strength shows no difference within the standard deviation of each matrix material. This allows us to reach the conclusion that the impregnation and the fibre/matrix interaction in fibre direction is identical and, therefore, the successful winding process can be reported. However, this test is set up to be dominated by the fibre strength and can only give insights into major failures of the winding process. To quantify fibre/matrix interaction and the interlaminar strength, an interlaminar shear stress test is performed.

### 5.2. Interlaminar Shear Strength Comparison of Composites with Thermosetting and Vitrimeric Matrices

**Figure 11:**
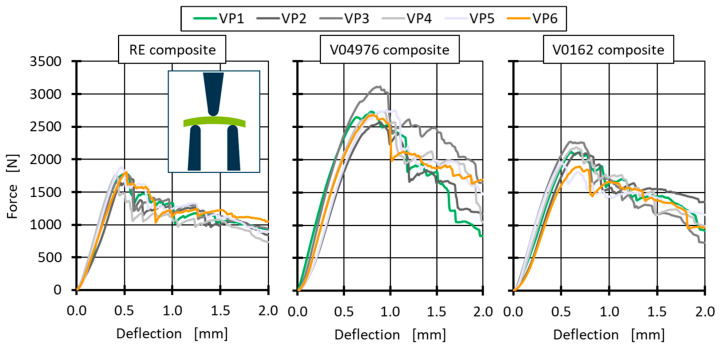
Force–deflection graphs of the ILSS measurements of all 3 matrix materials with 6 samples each. The graphs are recorded at room temperature. The pictogram shows the positioning of the curvature in the ILSS-tests.

The interlaminar shear strength (ILSS) test is designed to apply shear force directly into the interlaminar zone of a composite. The ILSS test is performed within a three-point bending test with a short sample of 20 ± 1 mm in length taken from additional ring segments with a width of 10 mm. In contrast to the DIN EN ISO 14130 [58], the samples are slightly curved due to being extracted from a pipe segment; the curvature is placed towards the middle fin of the three-point bending test (see pictogram in Figure 11). Pretesting force to straighten the sample is not applied, since a pretesting deflection of 0.34 mm would be needed, thus reducing the deflection until break by up to 69% (RE at 23 °C). Additionally, the samples vary in thickness (see Table 5) and, therefore, do not fulfil the 2 mm thickness requirement of DIN EN ISO 14130 [58]. However, an adjustment for sample thickness (d) and width (w) with the maximum force at the point of failure (F) is possible to calculate the interlaminar shear strength (τ) (Gl. 1).

(1) $$\tau =\frac{3}{4}\;\frac{F}{b*h}$$

To evaluate the matrix performance and to get a better understanding of the limits of vitrimeric materials, the ILSS test is performed at four different temperatures (23 °C, 80 °C, 100 °C and 140 °C), with the ultimate temperature 140 °C being close to T_g_ of RE and V04976 (see Table 3). The V0162 shows a slightly higher T_g_ so it can be expected to be the highest performing at 140 °C. It must be assumed that the maximum service temperature of 100 °C (~40 °C below the measured T_g_) should show identical mechanical behaviour for the vitrimeric material if it is to substitute the conventional thermoset matrix for the application in type-4 pressure vessels.

Individual ILSS tests are performed at each temperature with six samples and a distance-controlled three-point bending test with 1 mm/min testing speed on the Z150 from ZwickRoell GmbH & Co. KG (Ulm, Germany) with an additional 10 kN load cell installed for increased accuracy. The maximum force to determine the ILSS is taken at the first drop in force, at which point each sample shows an interlaminar failure (Figure 11). Throughout all resin-systems and samples, a brittle fracture is observed by a sharp drop in applied force after the maximum force is reached. Additionally, Figure 11 shows different deflections at the point of failure, where the RE composite shows the least deflection with the least amount of force absorbed before failure. Both vitrimeric samples are able to absorb more forces and break at higher deflection. This is in accordance with the uniaxial tests performed at the non-reinforced resin-systems and suggests a more ductile (thermoplastic-like) behaviour of the vitrimeric samples.

**Figure 12:**
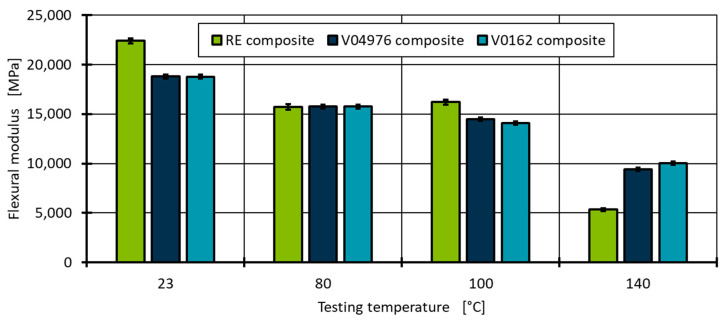
Composite flexural modulus at four different testing temperatures between 0.2 and 0.4 mm deflection of the RE in comparison to the vitrimeric matrix of CFRP pipe segments.

The flexural modulus is calculated from the slope between 0.2 and 0.4 mm deflection for each temperature to evaluate the initial composite response to an applied force. At room temperature (23 °C), the RE composite outperforms the composites with vitrimeric matrices by a ~16% higher flexural modulus (Figure 12). This is in accordance with the uniaxial tests with the non-reinforced resin-system. However, at elevated temperatures, the flexural modulus of the RE decreases, which is in contrast to the thermo-mechanical analysis of the non-reinforced specimens, to a point at which the composite behaviour is similar throughout all material combinations. As expected, at T_g_ (140 °C), the RE flexural modulus decreases drastically to 23.8%. However, the vitrimeric material combinations retain close to double the modulus, whereas the V0162 retains the highest flexural modulus, as suggested by the higher T_g_ (Figure 12). However, the V04976 composite retains 50.0% of its flexural modulus despite showing a similar T_g_ as RE in DSC measurements. This could be attributed to the vitrimeric properties allowing for the consolidation of pores under load and temperature, reaching higher localised fibre contents and, therefore, a higher flexural modulus. This is in contrast to expectations since the vitrimeric formulation allows for a rearrangement of the molecular network and, therefore, should show, in general, a lower matrix stiffness and, thus, a lower composite flexural modulus at all temperatures.

**Figure 13:**
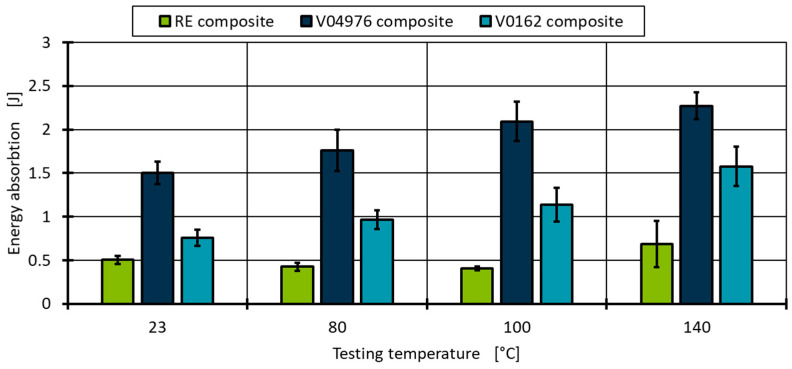
Evaluation of the energy absorbed during the ILSS tests at four temperatures and three material combinations.

The force at break of the vitrimeric matrix composites is even at higher room temperatures, which is shown in Figure 11. This contributes to the higher ductility of the vitrimeric matrix shown by the energy absorption before failure (Figure 13). The higher ductility allows for more uniform stress distribution within the composite and, thus, higher failure forces. The high stiffness and lack of molecular mobility of the RE lead to local stress concentration that cannot be dissipated and lead, therefore, to earlier failure, where the vitrimeric matrix can compensate for high localised forces by network morphology changes.

The most ductile behaviour is shown by the V04976 formulation, followed by the V0162 formulation. This is expected, since the EPIKOTE 0162 is a refined version of the EPIKOTE 04976 with sharp molecular weight distribution, resulting in a less ductile behaviour even in commercial thermoset applications [40].

**Figure 14:**
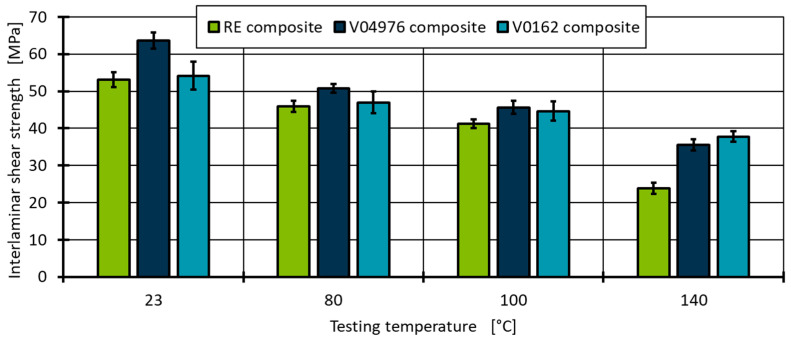
Interlaminar shear strength at four different testing temperatures of the RE in comparison to the vitrimeric matrix of CFRP ring segments.

The evaluation of the ILSS reveals the highest material performance for the V04976 (Figure 14). The higher ductility of the matrix material and the similar composite stiffness to the other matrix materials allow the applied force to be distributed within the sample, resulting in an overall better matrix performance in the ILSS test.

At 140 °C, both vitrimeric material combinations are within one standard deviation and cannot be distinguished significantly. The RE, as expected from the evaluation of ductility and composite stiffness, results in the lowest ILSS value. Based on these results, it is concluded that the composite performance of carbon fibre reinforced vitrimeric epoxy formulations using a 4-AFD curing agent show advanced material performance in comparison to a reference epoxy thermoset formulation. Even though a higher pore content is measured in the visual analysis of the vitrimeric composite, the composite performance is superior compared to the thermosetting reference material.

## 6. Discussion

The EPIKOTE 04976 epoxy resin is a material for wet filament winding and, in particular, for the manufacturing of type-4 pressure vessels for hydrogen storage. The epoxy resin in the commercially available conventional thermoset formulation (RE) is compared to two vitrimeric formulations (V04976 and V0162) using aromatic 4-aminophenyl-disulfide (4-AFD) as curing agent. All three material combinations are first characterised as non-reinforced resin systems using thermal characterisation and uniaxial tensile tests. Using DSC measurements, the glass transition temperature (T_g_) is determined to be 144 °C for RE and 138 °C for V04976, whereas the V0162 shows a much higher transition temperature at 158 °C. The difference in T_g_ relates directly to the higher retention of mechanical properties at 140 °C of 69.8% (V0162) to the 44.9% in the ILSS test. Since the tensile measurements of the not reinforced materials showed a significant elongation before break at temperatures > 80 °C for all materials, a similar behaviour in the application of hydrogen storage can be assumed. Additional transition temperatures such as the topology freezing temperature could not be identified below 230 °C using thermal analysis. This qualifies all materials for the temperatures experienced during the hydrogen loading process (<100 °C).

After the qualification of the matrix material, the process behaviour in an industrial-scale production process is demonstrated. Due to the crystalline form of the vitrimeric curing agent at room temperature with a melting temperature of 84 °C, the process had to be adjusted to an untypically high resin bath temperature of 80 °C with premixed resin and curing agents. This shows a need for industrial process adaptation to substitute for vitrimeric materials using the same epoxy resin successfully. All three materials were successfully processed using a wet filament winding process on an industrial-scale setup, winding six hoop layers with carbon fibre reinforcement, resulting in 500 mm long pipe-shaped samples. These hoop layers are representative of the type-4 pressure vessel since those layers are the load-bearing structure at the centre of the pressure vessel. The resulting composite quality is analysed by fibre content and porosity by visual inspection. The RE composite shows the lowest porosity and highest fibre content due to the higher viscosity of the vitrimeric material, even though a process adaptation of increased processing temperature by 20 °C was used. This shows that vitrimeric materials are processable by industrial processes; however, further process adaptation is necessary to achieve identical composite qualities to the reference epoxy material.

Due to a higher ductility of the vitrimeric matrix materials in the composite application at all temperatures, a superior ILSS is determined in comparison to the RE composites. This is in contrast to the finding of the neat materials since the elongation at break of the neat RE at 80 °C shows higher values. Within a composite, higher ductility allows for a distribution of stresses, resulting in a higher composite strength value. Therefore, the vitrimer matrix material could allow a better force distribution in a pressurised type-4 pressure vessel and reach even higher pressure values before failure compared to RE. Additionally, the self-healing properties of the vitrimer could lead to minor failure healing during the loading of the pressure vessel due to the temperature increase coupled with pressure application.

## 7. Conclusions and Outlook

Both vitrimeric formulations are processable by wet filament winding at industrial-scales, showing superior composite strength and ductility with comparable values for composite stiffness at maximum temperatures during hydrogen loading. The vitrimeric formulation, therefore, provides extended potential to replace the conventional thermoset formulation, introducing repair, recycle and reuse properties to type-4 pressure vessels for hydrogen storage. However, minor process adjustments are justified by the material properties of the precursor materials, such as the handling of the crystallinity of the vitrimeric curing agent and a non-conventional high processing temperature to reduce the viscosity during processing. To address the reuse and recycle characteristics of the vitrimeric type-4 pressure vessel, trials to unwind the here-produced pipes aim to repurpose much of the fibre length as possible in a different or the same product.

## Figures and Tables

**Table 1:** Material combinations used for the demonstration of an industrial-scale wet filament winding process.

Resin	Curing Agent	Catalyst	Abbreviation
55.3 wt % EPIKOTE 04976	44.2 wt % EPIKURE 04976	0.55 wt % EPIKURE Catalyst 04976	RE
69.9 wt % EPIKOTE 04976	30.1 wt % 4-AFD	-	V04976
69.9 wt % EPIKOTE 0162	30.1 wt % 4-AFD	-	V0162

**Table 2:** Glass transition temperatures evaluated from DSC-measurements according to DIN EN ISO 11357-1 of RE, V04976 and V0162.

Material	RE	V04976	V0162
T_g_	144 °C	138 °C	158 °C

**Table 3:** Test sequence of the vitrimer shaping tests with temperature, duration and additionally applied mass.

Material	Temperature [°C]	Test Duration	Test Weight
V04976	220 °C	1.5 h	Mould not weighted down
		1 h	185 g
		1 h	317 g
V0162	240 °C	2 h	Mould not weighted down
		1 h	185 g
		0.5 h	317 g
		0.5 h	609.5 g
		0.5 h	800 g
		1 h	1577 g

**Table 4:** Averaged strength for each material combination and the share of the theoretical fibre-only strength.

Matrix Material	Specimen Strength	Share of Fibre Strength
RE	4.46 ± 0.11 GPa	87.4%
V04976	4.49 ± 0.39 GPa	88.0%
V0162	4.58 ± 0.17 GPa	89.8%

**Table 5:** Average thickness measured using digital callipers and width of the ILSS samples averaged over 6 samples for each material combination.

Matrix Material	Average Thickness	Average Width
RE	2.45 ± 0.06 mm	10.08 ± 0.02 mm
V04976	3.20 ± 0.14 mm	10.11 ± 0.02 mm
V0162	2.81 ± 0.06 mm	10.12 ± 0.01 mm

## Data Availability

The original contributions presented in this study are included in the article. Further inquiries can be directed to the corresponding author.
